# The Cell Wall, Cell Membrane and Virulence Factors of *Staphylococcus aureus* and Their Role in Antibiotic Resistance

**DOI:** 10.3390/microorganisms11020259

**Published:** 2023-01-19

**Authors:** Philip Nikolic, Poonam Mudgil

**Affiliations:** School of Medicine, Western Sydney University, Campbelltown, NSW 2560, Australia

**Keywords:** *Staphylococcus aureus*, infectious diseases, antibiotic resistance, MRSA, cell wall, cell membrane lipids, virulence factors

## Abstract

Antibiotic resistant strains of bacteria are a serious threat to human health. With increasing antibiotic resistance in common human pathogens, fewer antibiotics remain effective against infectious diseases. *Staphylococcus aureus* is a pathogenic bacterium of particular concern to human health as it has developed resistance to many of the currently used antibiotics leaving very few remaining as effective treatment. Alternatives to conventional antibiotics are needed for treating resistant bacterial infections. A deeper understanding of the cellular characteristics of resistant bacteria beyond well characterized resistance mechanisms can allow for increased ability to properly treat them and to potentially identify targetable changes. This review looks at antibiotic resistance in *S aureus* in relation to its cellular components, the cell wall, cell membrane and virulence factors. Methicillin resistant *S aureus* bacteria are resistant to most antibiotics and some strains have even developed resistance to the last resort antibiotics vancomycin and daptomycin. Modifications in cell wall peptidoglycan and teichoic acids are noted in antibiotic resistant bacteria. Alterations in cell membrane lipids affect susceptibility to antibiotics through surface charge, permeability, fluidity, and stability of the bacterial membrane. Virulence factors such as adhesins, toxins and immunomodulators serve versatile pathogenic functions in *S aureus*. New antimicrobial strategies can target cell membrane lipids and virulence factors including anti-virulence treatment as an adjuvant to traditional antibiotic therapy.

## 1. Introduction

Antibiotic resistance is a serious threat in the clinic with a variety of infectious bacteria possessing resistance to commonly used antibiotics. Of particular concern is methicillin resistant *Staphylococcus aureus* (MRSA). MRSA has developed resistance to antibiotics including penicillin and methicillin with some strains having developed additional resistances to daptomycin and vancomycin, complicating treatment further [1,2]. This rapid increase in antibiotic resistance has outpaced the development of new antibiotic compounds and threatens to end the golden age of antibiotics and lead to the start of the post-antibiotic era. As such, research has been directed towards developing novel treatments outside of traditional antibiotics as well as towards a deeper understanding of the changes that occur in bacteria after they develop antibiotic resistance.

The cell wall, cell membrane and virulence factors of *S. aureus* are important components necessary for its survival and ability to infect humans. The cell wall of *S. aureus* consists of a layer of peptidoglycan and is responsible for maintaining cell shape and resisting osmotic pressure [3]. The cell membrane in *S. aureus* is a phospholipid bilayer that acts as a selectively permeable barrier and protects the cell from the entry of harmful substances such as antibiotics [4]. Virulence factors are molecules that assist the bacterium in establishing an infection, damaging the host and interfering with host immunity [5]. These cellular components represent current and potential targets for antibiotics and other novel treatments. A deeper understanding of them and how they change in antibiotic resistant *S. aureus* is essential for the continued development of effective antibacterial treatment. This review presents an overview of antibiotics and development of antibiotic resistance in *S. aureus* as well as characteristics of the bacterial cell wall, cell membrane, and virulence factors and how these change in resistant strains.

## 2. Antibiotics

Antibiotics are a class of compounds that can inhibit the growth of or kill bacteria. The first and most famous antibiotic to be discovered was penicillin. However, even before the discovery of penicillin, other non-antibiotic antimicrobials had been discovered and used. In 1909, arsphenamine was discovered by Sahachiro Hata and was used to treat syphilis and trypanosomiasis in 1910. It remained the most used antimicrobial drug until the introduction of antibiotics in the 1940s [6].

**Figure 1:**
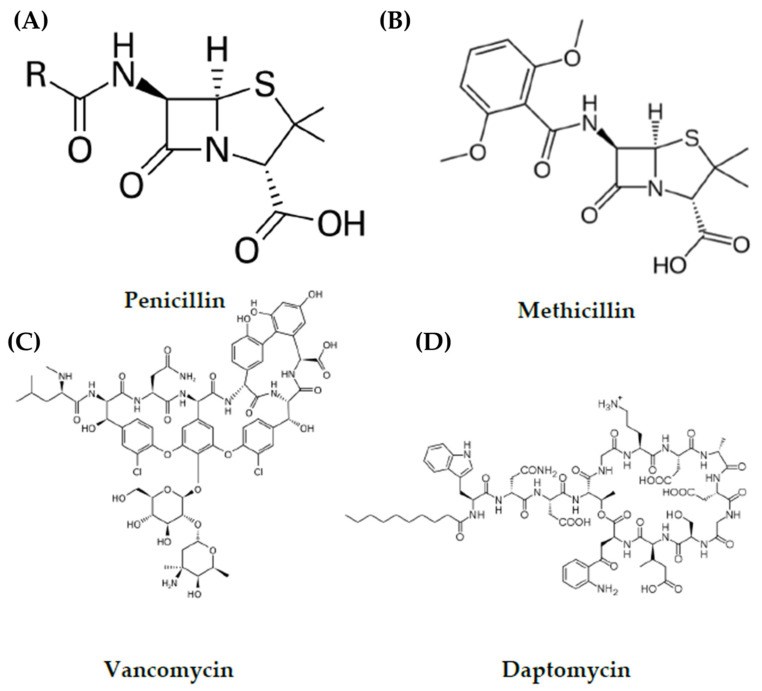
Structure of antibiotics used against *S. aureus*. (**A**) Penicillin G. (**B**) Methicillin. (**C**) Vancomycin. (**D**) Daptomycin.

Penicillin (Figure 1A) was discovered by Alexander Fleming in 1928 after he noticed that an old culture plate (contaminated with *Penicillium notatum*) grew a mould that prevented the growth of *S. aureus*. He experimented with different moulds to better understand this effect and determined that not all moulds produced the antimicrobial component detected on this plate. While he failed to isolate the active molecule itself, he named it penicillin. In 1940 Howard Florey and Ernst Chain published a paper that described a purification technique for penicillin and in 1945 penicillin was available for limited use in human treatment [7]. 

Even before the discovery of penicillin by Alexander Fleming in 1928, mould had been recognised as having medicinal properties. In ancient Egypt, Greece, China and Rome topical applications of mouldy bread were used to treat infections. In 1870, Sir John Scott Burdon-Sanderson described mould covered culture fluid as being capable of inhibiting bacterial growth and in 1871 Joseph Lister conducted experiments using *Penicillium glaucium* showing an antibacterial effect [7]. While other scientists may have discovered the antibacterial properties of mould it was only when Fleming’s work brought more attention to these properties that the golden age of antibiotics began.

Penicillin and all members of the penicillin class of antibiotics are derivatives of 6-aminopenicillanic acid with a β-lactam ring structure responsible for their antimicrobial activity. They work by attaching to enzymes called penicillin binding proteins (PBPs) that play a key role in the synthesis of the peptidoglycan cell wall. The binding of penicillin to these PBPs inhibits peptidoglycan cross link formation in bacterial cell wall synthesis. Both modification to PBPs and the presence of enzymes that can cleave the β-lactam ring structure are known resistance methods found in bacteria. In fact, by 1957, more than 80% of hospitals reported the presence of penicillin resistant strains of *S. aureus*. It was this resistance that prompted more research into alternative antibiotics. By the end of the 1950s semi-synthesis around the 6-aminopenicillanic acid core of penicillins presented a new class of penicillinase resistant penicillins including methicillin (Figure 1B). Prior to this in 1943, streptomycin was isolated by Selman Waksman from the soil bacterium *Streptomyces griseus* [6]. Streptomycin was the first antibiotic available for use against tuberculosis. Waksman also established that as many as 50% of actinomycetes in soil were capable of antimicrobial activity [6]. This thereby directed research towards the discovery of naturally produced antibiotics in soil bacteria.

## 3. Antibiotic Resistance

Bacteria that can grow even in the presence of antibiotics are said to be antibiotic resistant. Whether a bacterial species is considered sensitive to or resistant to an antibiotic depends on the minimum inhibitory concentration (MIC) breakpoint which is the concentration of an antibiotic that represents the barrier between resistance and sensitivity. If the concentration needed to kill the bacterial strain is less than or equal to the MIC breakpoint, that strain is considered susceptible to that antibiotic. If the amount required is higher, it is considered resistant [8]. The MIC breakpoint is set based on the highest safe level of the drug in human serum when administered [9]. 

**Figure 2:**
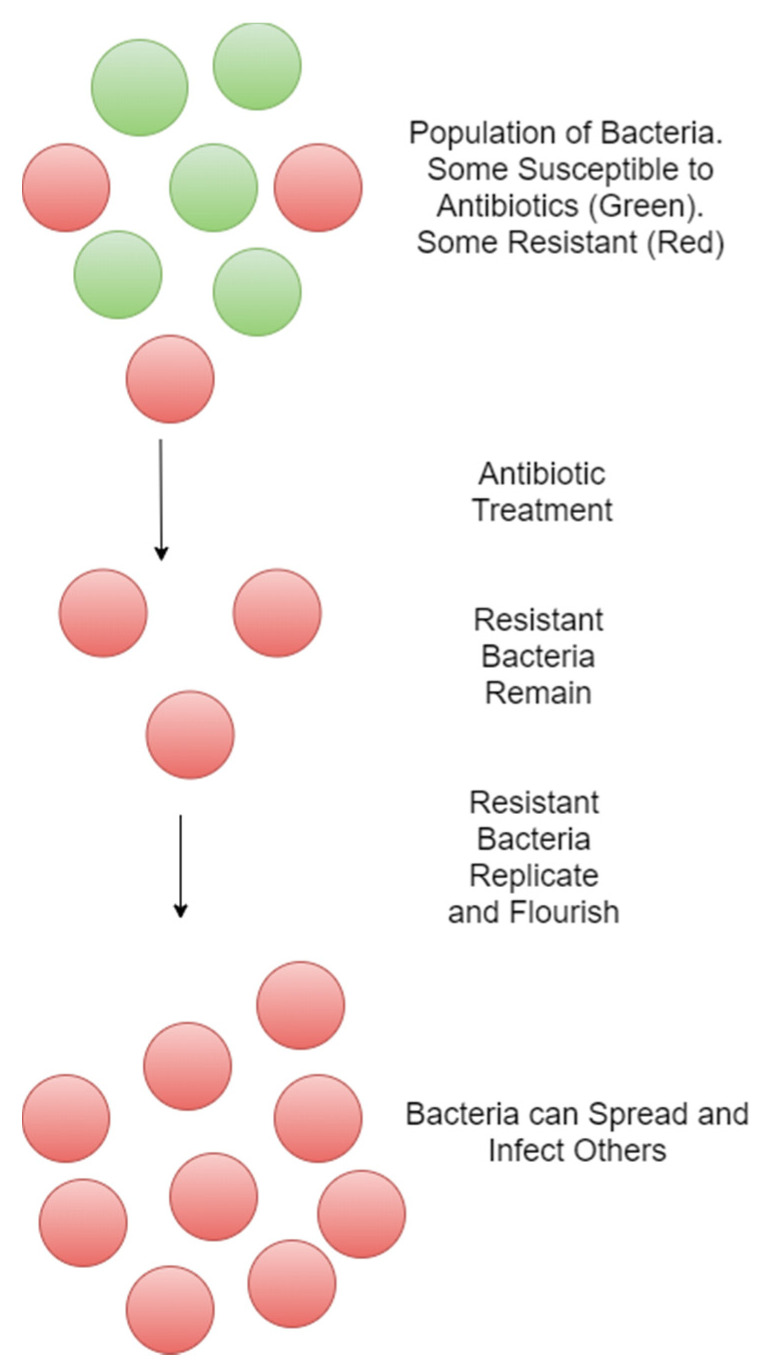
Spread of antibiotic resistant bacteria by natural selection.

The rate of antibiotic resistance development currently exceeds the rate at which new drugs are developed. This threatens to put an end to the golden age of antibiotics and lead to the start of the post-antibiotic era. This would have a far reaching effect on medical procedures as they all rely on the use of antibiotics to manage infections [10]. Many antibiotics are derived from natural products that are used as defence mechanisms by bacteria against other bacteria [8]. Therefore, it is only expected that resistance to these products would also be found naturally in the environment as otherwise the bacteria that produce these molecules as a defence mechanism would also be killed by them. However, the increased use of these natural products as therapeutic agents has created a stronger selection pressure than would normally be seen and has thus promoted the increased development and growth of antibiotic resistant bacteria (Figure 2). This is thought to occur both due to random mutation and horizontal gene transfer [11]. Furthermore, the exposure of bacteria to environmental stresses including sub-inhibitory concentrations of antibiotics and bacteriophages can promote the induction of antibiotic resistance through mechanisms that increase the mutation rate [12]. One such bacterial species that has readily developed resistance and become a significant problem in clinical settings is *S. aureus*.

## 4. *Staphylococcus aureus*

*Staphylococcus aureus* is a Gram-positive cocci bacterium that can be found as a common commensal on human skin and in the human nasal cavity [13]. There are also reports of *S. aureus* being present in the oropharynx [14]. Around 30% of the population live asymptomatically colonised with *S. aureus* [13]. However, *S. aureus* can also act as a pathogenic bacterium and is the cause of a variety of human infectious diseases that range from skin infections such as impetigo to more serious conditions such as osteomyelitis and endocarditis [14]. Infections with *S. aureus* are typically treated with antibiotics but the increased development and spread of antibiotic resistant strains has made treatment increasingly difficult [15]. 

### Methicillin Resistant Staphylococcus aureus

Methicillin resistant *S. aureus* is a widely studied antibiotic resistant strain that is of clinical significance. As many as 60% of clinically isolated strains of *S. aureus* have been found to be resistant to methicillin [3].

There exist three kinds of MRSA infections that are characterized by the origin of the infection. The first is called healthcare-associated MRSA (HA-MRSA) and occurs when someone is infected with MRSA in a healthcare setting (such as a hospital or nursing home) [13]. This is typically through surgery or the use of catheters that compromise the body and provide a portal of entry for bacteria into the body [16]. This is made more dangerous by the individual usually being immunocompromised. HA-MRSA is associated with blood stream infections and pneumonia [13]. 

The second kind of MRSA infection occurs in the wider community and usually affects healthy individuals that have not recently been in contact with a healthcare facility [17]. This is called community-associated MRSA (CA-MRSA). This form of MRSA infection is associated with skin and soft tissue infections and is usually spread by skin contact [13]. CA-MRSA infections were initially defined by a set of criteria. This excluded healthcare risks such as a prolonged hospital stay or need for haemodialysis [16]. Furthermore, it included hospitalised patients if the bacterium was isolated within 48 h of admission [16] (Table 1). CA-MRSA strains have also been identified by molecular characteristics such as pulsed field gel electrophoresis (PFGE) profile, multilocus sequence type (MLST), staphylococcal protein A (*spa*) type and staphylococcal chromosomal cassette *mec* (SCC*mec*) type [16]. 

These categories of MRSA were the only two thought to exist until 2004 when an MRSA strain was found colonising the family of a pig farmer in the Netherlands [18]. This strain was different from those typically identified in HA-MRSA and CA-MRSA and was also found in a pig present on the farm. This helped to demonstrate that MRSA could be transmitted between animals and humans and was, along with other strains with a similar presentation, termed livestock-associated MRSA (LA-MRSA) [18]. 

HA-MRSA and CA-MRSA are genetically distinct, with HA-MRSA resistant to multiple antibiotic classes but CA-MRSA only resistant to β-lactams and macrolides [13]. HA- MRSA was initially the most common form of MRSA infection; however, this has changed in recent years with CA-MRSA becoming more common than HA-MRSA in some regions including in Australia [19] and in children in some regions of the United States [17]. 

Furthermore, CA-MRSA accounts for 70% of all community-associated infections with staphylococci and 74% of all *S. aureus*-caused pneumonia at Texas Children’s Hospital [10]. Cases of HA-MRSA strains infecting people in the community and CA-MRSA infecting hospital patients have, however, begun to obscure the line between CA-MRSA and HA-MRSA and have made many of the methods of distinguishing the two invalid.

LA-MRSA by comparison is less of a threat to human health. LA-MRSA only account for 3.9% of MRSAs isolated in the European Union and typically only cause lower severity infections [18]. Despite this, it should be acknowledged that any *S. aureus* strain can become a life-threatening strain and so the impact of LA-MRSA must not be ignored [18].

One of the most common clonal complexes of LA-MRSA is CC398. Evidence suggests that this isolate initially arose from MSSA CC398 in humans that infected livestock where it later acquired SCC*mec* to develop methicillin resistance [20]. This was the clonal complex isolated from the pig and pig farmer in 2004 [18]. Other clonal complexes have also been associated with LA-MRSA including CC9/t1430 and CC5/t002 which accounted for almost 27% of MRSA isolated from chicken meat and 10% of MRSA isolated from meat in Germany [20]. 

Interestingly, LA-MRSA (particularly CC398) possesses a multiresistance phenotype like HA-MRSA. CC398 has resistance to oxacillin, erythromycin, clindamycin and oxytetracycline [20]. Typically, infection with LA-MRSA in livestock is asymptomatic but infection in humans can lead to skin and soft tissue infections [21]. 

## 5. Development of Antibiotic Resistance in *Staphylococcus aureus*

**Figure 3:**
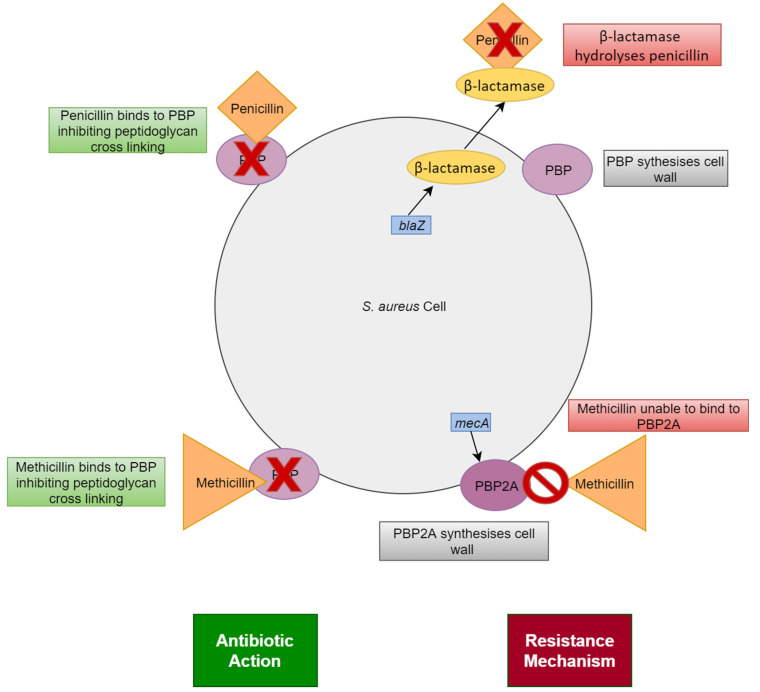
Antibiotic action of penicillin and methicillin against *S. aureus* and resistance mechanisms used by *S. aureus* against penicillin and methicillin.

Penicillin was discovered in 1928 but it was not introduced for medical use until 1944 [22]. Once used in the clinic however, it allowed for the management of serious bacterial infections that were previously unresponsive to treatment, including those of Staphylococci. In 1946, shortly after its introduction, the first penicillin resistant *S. aureus* was isolated [23]. These penicillin resistant strains were resistant due to the acquisition of the *blaZ* gene carried on Tn552-like transposons or the remnants of these transposons [8]. The *blaZ* gene codes for a protein called β-lactamase (also known as penicillinase) that inhibits the action of penicillin by hydrolysing the β-lactam ring structure [8] (Figure 3). Penicillin resistance is defined by an MIC of ≥0.25 mg/L [24].

After penicillin resistance was identified in *S. aureus*, an alternative antibiotic treatment was needed. This came in the form of methicillin which was introduced in 1959 to treat penicillin resistant strains, but, much like with penicillin, the indiscriminate use of this antibiotic promoted the development and spread of resistance and in 1961 (only two years after its introduction) there were cases of strains of methicillin resistant *S. aureus* reported in England [25]. Other countries soon reported similar resistance [22]. Methicillin resistance is defined by an MIC of oxacillin ≥ 4 mg/L [26].

*S. aureus* gained resistance to methicillin by acquisition of the *mecA* gene present on the mobile genetic element SCC*mec* [25]. There exist 15 types of SCC*mec* (from I to XV) based on different combinations of five *mec* complexes and nine *ccr* complexes [27]. SCC*mec* Types XIV and XV were only recently characterized in 2019 and 2022, respectively [27,28]. This highlights the ever-changing nature of MRSA and the need for continued monitoring of antibiotic resistant bacteria. SCC*mec* is integrated into the *S. aureus* genome at the same point, an *attB* integration site sequence at the 3′ end of the *orfX* gene [29]. The *mecA* gene codes for a protein called penicillin binding protein 2A (PBP2A) that can cross-link peptidoglycan after the native penicillin binding proteins are inactivated by β-lactam antibiotics (such as methicillin) [30] (Figure 3). While β-lactam antibiotics such as methicillin can bind to PBP2A, a distorted active site means that the antibiotics cannot inactivate the protein thereby rendering their antibacterial activity ineffective [30,31].

The acquisition of the SCC*mec* and the subsequent evolution of MRSA is something that is still being investigated. Two hypotheses have been proposed to explain the emergence and spread of MRSA in hospitals and the community. The first is the single-clone theory that suggests all MRSA clones have a common methicillin sensitive *S. aureus* (MSSA) ancestor [32]. The second, the multi-clone theory, suggests that the SCC*mec* containing *mecA* was introduced several times to different ancestral MSSA strains [32]. The multi-clone theory is better supported by the literature. For example, in Enright et al. [22] several SCC*mec* types were identified in different strains of MRSA. Furthermore, there are more genetic differences between MRSA strains than would be expected if they all originated from a single MSSA strain [22,32]. This supports the independent development of MRSA from multiple different MSSA strains. 

The origin of the SCC*mec* found in MRSA is unknown but it is found in other species of coagulase negative staphylococci (named such to distinguish from the coagulase producing *S. aureus*) [33]. It is speculated that SCC*mec* is capable of being transferred between staphylococci species and the literature considers the most likely origin to be from a coagulase negative staphylococcus [33]. Two of the more likely candidates are *Staphylococcus sciuri* and more recently *Staphylococcus fleuretti* [29].

Vancomycin (Figure 1C) was considered one of the last resort antibiotics for treating MRSA, but even this has been rendered ineffective against certain strains of MRSA that have acquired vancomycin resistance [34]. Initially, isolates of MRSA with reduced susceptibility to vancomycin were reported in 1997 [2]. This was either as vancomycin insensitive *S. aureus* (VISA) or heterogeneous VISA (hVISA). VISA refers to strains with reduced susceptibility in the entire population whereas hVISA refers to populations with acceptable MIC of vancomycin that also contain a small subpopulation (around one in every 10^5^–10^6^ cells) that have reduced susceptibility to vancomycin [2,34]. VISA is typically caused by chromosomal mutations that confer slightly increased resistance to vancomycin [2]. In addition, there exist strains of vancomycin resistant *S. aureus* (VRSA). The first vancomycin resistant strain of *S. aureus* was reported in the US in 2002 [34]. Vancomycin resistance is defined as an MIC ≥ 16 mg/L and is found in strains of MRSA due to the acquisition of the *vanA* operon unlike in VISA [35]. This operon is present on the Tn1546 transposon and originally came from the vancomycin resistant enterococci (VRE) conjugative plasmid [35,36].

**Figure 4:**
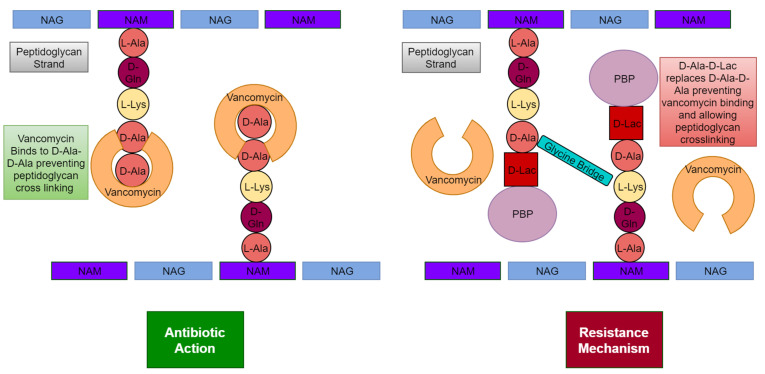
Antibiotic action of vancomycin against *S. aureus* and resistance mechanisms used by *S. aureus* against vancomycin.

The *vanA* operon confers vancomycin resistance by altering the synthesis of the cell wall to remove the vancomycin binding site (Figure 4). The cell wall in Gram-positive bacteria is made up of cross-linked peptidoglycan strands [35]. These strands are made up of glycan chains of N-acetylglucosamine (NAG) and N-acetylmuramic acid (NAM), cross linked by glycine bridges. Vancomycin interferes with peptidoglycan synthesis by binding to the final D-Ala-D-Ala residue of the newly synthesised peptide and disrupting downstream assembly [35]. However, in VISA the proteins VanA, VanH, VanX and VanY coded by the *vanA* operon remove the vancomycin binding site. This is done in two major steps. In the first, the dipeptide D-Ala-D-Ala peptidoglycan precursors are hydrolysed by VanX (a D,D-dipeptidase) and VanY (a D,D-carboxylpeptidase). In the second major step, VanA (a ligase that catalyses ester bond formation) and VanH (a dehydrogenase that forms D-Lac by reduction of pyruvate) synthesise D-Ala-D-Lac (that vancomycin cannot bind) to replace the D-Ala-D-Ala residue [35].

**Figure 5:**
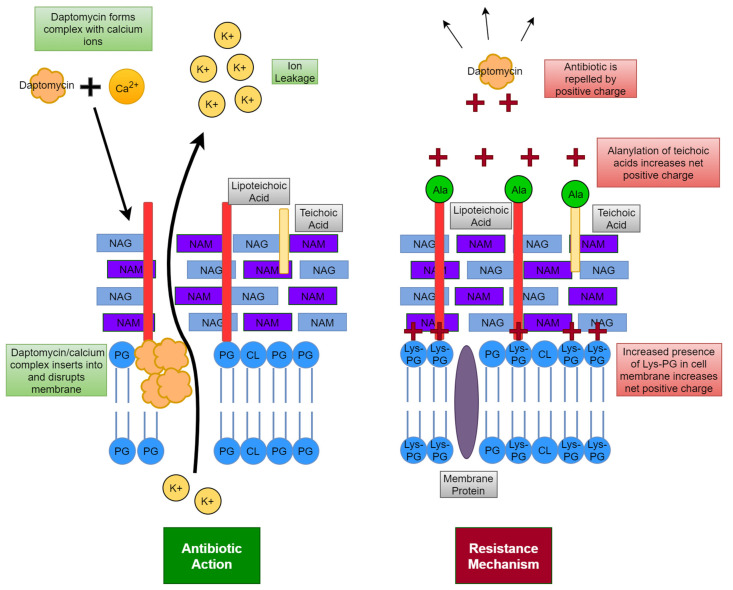
Antibiotic action of daptomycin against *S. aureus* and resistance mechanisms used by *S. aureus* against daptomycin.

**Figure 6:**
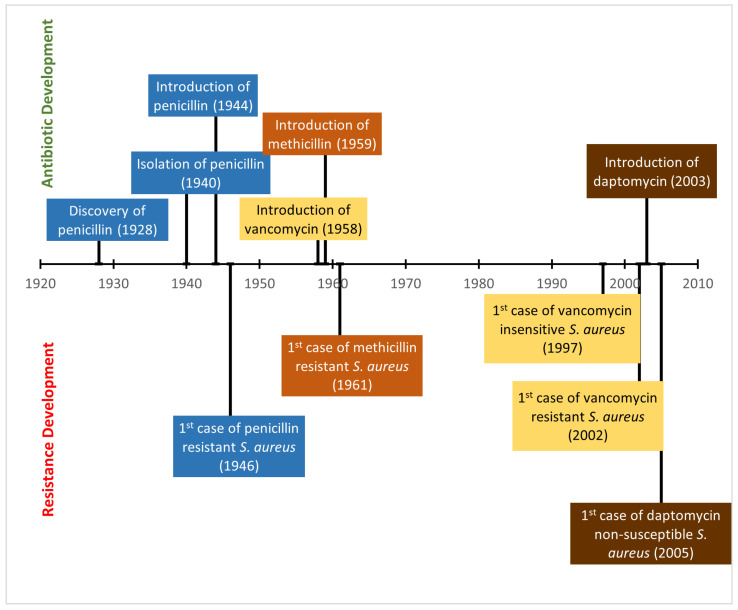
Timeline of development of antibiotic resistance in *S. aureus*.

Daptomycin (Figure 1D) is a cyclic peptide antibiotic. It has been approved for use against *S. aureus* and is considered a mainstay of anti-MRSA therapy [37]. Daptomycin’s mode of action requires calcium. Daptomycin and calcium form a complex that behaves as a cationic peptide and oligomerises to form micelles [8]. These micelles then penetrate the cell wall and insert into the lipid membrane by binding to phosphatidylglycerol. The micelles thereby disrupt the membrane causing depolarisation, permeabilisation and ion leakage [8]. An MIC breakpoint that determines resistance has not been determined yet for daptomycin in *S. aureus* but a level for susceptibility has been set. An MIC of <1 mg/L is considered susceptible for bacteria and an MIC ≥1 mg/L is considered non-susceptible [1]. There are several potential explanations for daptomycin non-susceptibility. These include a more positively charged membrane, changes to membrane fluidity and alanylation of teichoic acids (Figure 5) [1]. The year of discovery of penicillin, methicillin, vancomycin and daptomycin can be seen in Figure 6 as can the year of the first reported development of resistance to these antibiotics in *S. aureus*.

## 6. Bacterial Cell Components

### 6.1. Cell Wall

**Figure 7:**
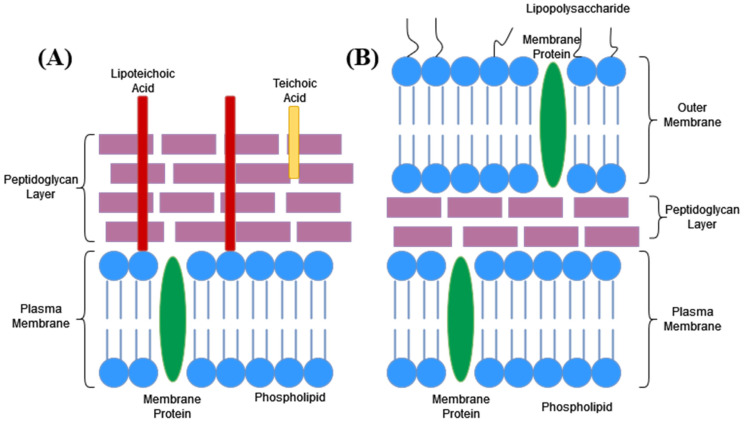
Bacterial cell wall structure. (**A**) Gram-positive bacteria. (**B**) Gram-negative bacteria.

**Figure 8:**
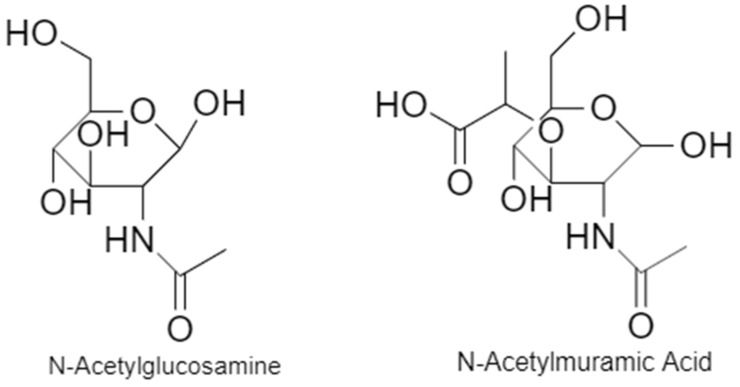
Structure of N-acetylglucosamine and N-acetylmuramic acid.

The bacterial cell wall is a key component of bacteria. Its roles include maintaining cell shape and allowing the cell to resist osmotic pressure [38]. The structure of the cell wall differs between Gram-positive bacteria and Gram-negative bacteria (Figure 7). Gram-positive bacteria have a thicker peptidoglycan layer and teichoic acids in their cell wall whereas Gram-negative bacteria have a thinner peptidoglycan layer and an outer membrane exterior to the peptidoglycan layer that contains lipopolysaccharides [39]. The peptidoglycan layer of Gram-positive bacterial cell walls makes up the majority of the layer and provides structural stability to the cell wall [3]. The peptidoglycan layer is made up of glycan strands that are polymerised and cross linked together by the collective action of more than 10 proteins [40,41]. These glycan strands are made up of alternating NAG and NAM residues (Figure 8). In addition to NAG and NAM, the glycan strand also includes a stem and a bridge component [41]. In *S. aureus* the stem component is the pentapeptide, L-Ala-D-iso-Gln-L-Lys-D-Ala-D-Ala and the bridge component is a pentaglycyl segment [41]. The disaccharide NAG and NAM are polymerised into the glycan chain (connected by a β(1–4) glycosidic bond) and the chain is then cross-linked to other chains between the N-terminus of the terminal glycine of the pentaglycyl segment attached to one stem, and the D-Ala of a neighbouring pentapeptide stem [41]. Penicillin binding proteins catalyse both the strand polymerisation (transglycosylation) and cross-linking (transpeptidation) steps in cell wall synthesis [40].

Glycan strands are modified after their synthesis and this modification differs between bacterial species [42]. One such modification found in *S. aureus* is O-acetylation. O-acetylation of NAM residues but not NAG residues has been observed in *S. aureus* [42]. The gene OatA was first identified in *S. aureus* and is responsible for synthesis of the O-acetyltransferase that catalyses the O-acetylation reaction of peptidoglycan [42]. This modification contributes to the resistance of pathogenic Gram-positive bacteria to lysozyme [42]. The teichoic acids present in the cell wall and the cross linking of the peptidoglycan layer also contribute to lysozyme resistance [42]. The amount of cross-linking found in the strands varies depending on the bacteria, with staphylococci generally having a high proportion of cross-linking (80–90%) [43]. The staphylococcal peptidoglycan layer ranges from 20 nm to 40 nm in thickness [3,44] and can vary significantly between *S. aureus* strains [45]. Glycan strands vary in chain length in different species, but this variation does not impact cell wall thickness [44]. In *S. aureus* the length of glycan strands is on average 18 disaccharide units with a majority being between 3–10 disaccharide units and about 10–15% being more than 26 disaccharide units [46].

**Figure 9:**
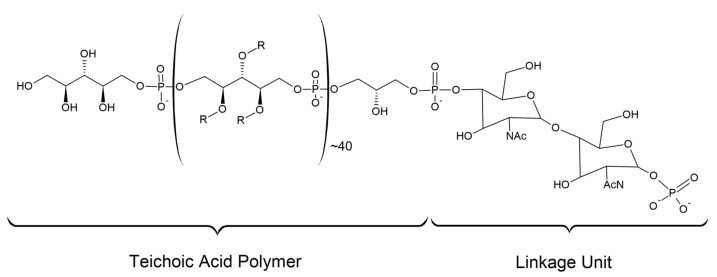
Structure of the cell wall teichoic acid of *S. aureus*. R=H, α-GlcNAc, β-GlcNAc, D-Ala.

Teichoic acids are found in the cell wall of Gram-positive bacteria, and in *S. aureus* it is estimated that every ninth peptidoglycan NAM residue possesses an attached teichoic acid [47]. Teichoic acids are anionic glycopolymers that extend beyond the peptidoglycan layer [48]. The structure of a wall teichoic acid (WTA) can be broken down into two main components [49]. The first is a disaccharide linkage unit. The disaccharide linkage unit contains an N-acetylmannosamine (β1→4) N-acetylglucosamine-1-phosphate (disaccharide) with one to three glycerol-3-phosphate (G3P) units attached to the C4 hydroxyl of the N-acetylmannosamine residue (linkage unit) [49,50]. The phosphate of the linkage unit is covalently attached to the C6 hydroxyl of a peptidoglycan NAM by a phosphodiester bond. The second component is the main chain polymer of phosphodiester linked polyol repeats. These repeats extend from the G3P end of the disaccharide linkage unit [49,50]. In *S. aureus* the polyol units are poly(ribitol-phosphate) repeats [51]. The structure of *S. aureus* WTAs is summarised in Figure 9 [51]. Teichoic acids play a variety of important roles in Gram-positive bacteria. They assist in host interaction, defining cell shape, antibiotic resistance and help protect the cell from turgor pressure [3,51]. In addition to teichoic acids, the cell wall also contains surface proteins. These proteins can be covalently linked to the peptidoglycan or non-covalently bound to cell wall polymers [46].

### 6.2. Cell Membrane

The bacterial cell membrane is a phospholipid bilayer and an essential component of the cell [52]. It acts as a selectively permeable barrier to maintain appropriate concentrations of nutrients and waste inside the cell. It is also crucial for proton motive force, generation of ATP, protection from the entry of harmful substances and cell to cell communication as it is the site of these processes [4]. In addition to phospholipids the cell membrane also contains membrane associated proteins that assist with these roles [4]. 

**Figure 10:**
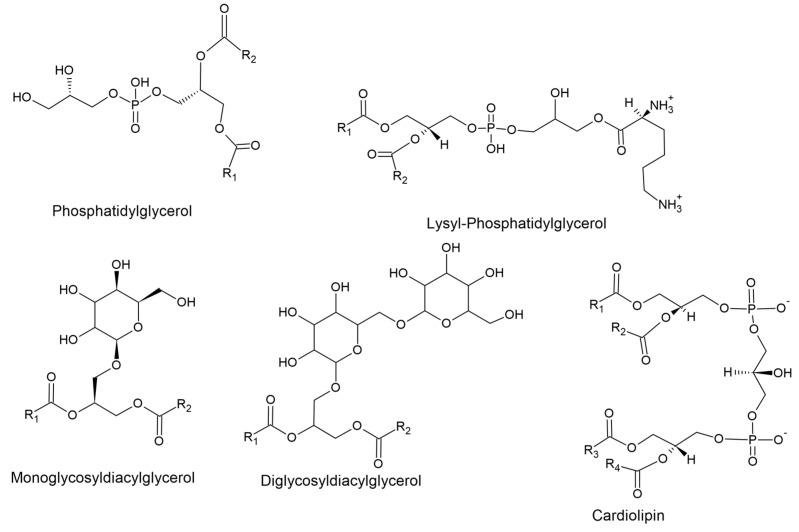
Structure of bacterial membrane lipids.

The bacterial cell membrane is made up of a variety of different species of lipids, including phospholipids and glycolipids [4,52]. Some of the lipids found in the membrane of *S. aureus* include phosphatidylglycerols (PG), lysyl-phosphatidylglycerols (Lys-PG) monoglycosyldiacylglycerols (MGDG), diglycosyldiacylglycerols (DGDG) and cardiolipins (CL) (Figure 10).

#### 6.2.1. Membrane Lipid Synthesis

**Figure 11:**
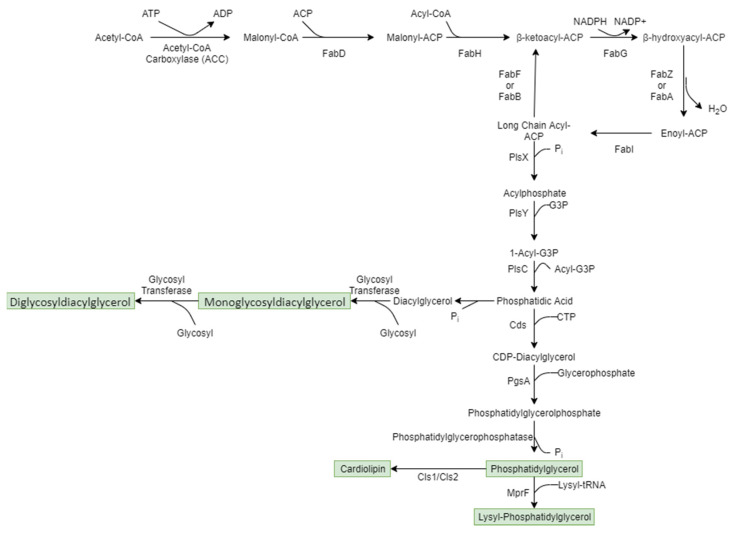
Cell membrane lipid synthesis pathway.

The first step in bacterial membrane lipid synthesis is the type II fatty acid biosynthetic pathway which takes place in the cytoplasm [53]. This pathway is summarised in Figure 11. The first step of type II fatty acid synthesis involves the action of acetyl-CoA carboxylase (ACC) that converts acetyl-CoA to malonyl-CoA with biotin as a cofactor. Next, the malonyl group is transferred to the acyl-carrier protein (ACP) by FabD (a malonyl translocase) forming malonyl-ACP. FabH then condenses another acyl-CoA with malonyl-ACP to form the first β-ketoacyl- ACP intermediate of the fatty acid synthase II pathway. Straight and branched chain fatty acids are then produced as part of the elongation step of the fatty acid synthase II pathway. The branched chains are introduced during the initiation phase by FabH enzymes. In *S. aureus* the FabH enzyme prefers larger branched-chain substrates [53]. 

Chain elongation is then carried out as four enzymatic reactions. At the end of these four steps, the growing acyl chain is advanced by two carbons. First the β-ketoacyl-ACP intermediate is reduced by the FabG β-ketoreductase producing β-hydroxyacyl-ACP which is then dehydrated by FabA or FabZ to enoyl-ACP. The cycle must then be pulled to completion by an enoyl-reductase (FabI). A new round of elongation is then initiated by an elongation condensing enzyme, either FabF or FabB and the cycle continues [53]. 

Staphylococcal phospholipid synthesis then starts by the acylation of G3P with the fatty acids. PlsX transfers the acyl group from the long chain acyl-ACP produced during the elongation step described above to inorganic phosphate to form the acylphosphate intermediate. PlsY then uses the acyl-phosphate as a substrate to acylate G3P to 1-acyl- G3P. A second fatty acid is then transferred from acyl-ACP to 1-acyl-G3P by the action of PlsC to create phosphatidic acid. Phosphatidic acid is the universal bacterial phospholipid precursor. The phosphatidate cytidylyltransferase Cds catalyses the synthesis of CDP-diacylglycerol from phosphatidic acid and cytidine triphosphate. PgsA then catalyses the generation of phosphatidylglycerolphosphate by replacing cytidine monophosphate with glycerolphosphate. Finally, phosphatidylglycerolphosphate is dephosphorylated by a phosphatidylglycerophosphatase to create phosphatidylglycerol [53]. The phosphatidylglycerol can then be modified to create other membrane phospholipids. It can be aminoacylated with an L-lysine group from a lysyl-tRNA by the multipeptide resistance factor (MprF) to create lysyl-phosphatidylglycerol [54]. It can also be converted into cardiolipin by the CL synthases Cls1 and Cls2. These enzymes condense two PG molecules into one CL and glycerol [55]. 

Alternatively, phosphatidic acid can be dephosphorylated to form diacylglycerol (DAG) as part of the synthesis of MGDG and DGDG [53]. After dephosphorylation of phosphatidic acid to DAG, glycosyl transferases then transfer a glycosyl residue to the DAG to form MGDG. The MGDG is then used as a substrate and a second glycosyl residue can be added to form DGDG [56]. One enzyme is responsible for the first glycosylation of the DAG, creating MGDG and another for the second glycosylation, creating DGDG [57]. The glycosyl transferase activity seen in *S. aureus* is limited to the cytoplasm side of the membrane with the final DGDG product being translocated to the extracellular side [56]. The enzymes responsible for the formation of MGDG and DGDG in *S. aureus* are part of the GT28 family [58].

#### 6.2.2. Role of Membrane Lipid Species

Bacterial cell membrane lipids can play important roles in antibiotic resistance and this role varies based on lipid class (Table 2). 

Phosphatidylglycerol is a phospholipid that contains two acyl chains, esterified to a glycerol which is then in turn bonded to a head group containing a phosphate [59] (Figure 10). Due to the negative phosphate and no other compensating positive charges, PG contributes an overall negative charge to the bacterial membrane [59]. Furthermore, PG functions as a stabiliser and destabiliser by strong electrostatic interactions between charged species [60]. This is believed to play an important role in controlling interactions between membrane peptides and proteins [60].

Lysyl-phosphatidylglycerol is a PG that has been aminoacylated with an L-lysine group from a lysyl-tRNA by the MprF protein [54]. The MprF protein consists of a hydrophilic C-terminal domain and hydrophobic N-terminal domain. The C-terminal domain is responsible for lysinilation of PG in the inner leaflet of the bacterial membrane whereas the N-terminal domain is responsible for translocation of Lys-PG across the membrane [54]. Lys-PG production has been shown to decrease *S. aureus* susceptibility to cationic antimicrobial peptides (CAMPs) by increasing cell surface charge [54]. Lys-PG has also been shown to play a role in cell cycle regulation by regulating DNA replication initiation [61]; this is through the DNA replication initiator protein DnaA which is inhibited by PG and CL promoting the release of ATP from it [61]. Lys-PG however, counteracts this interaction, which could be due to a decrease in the negative charge of the membrane or interruption of acidic phospholipid cluster domains as a result of increased presence of Lys-PG [61]. 

Cardiolipin is made up of four acyl chains and subsequently has a large number of potential species [62]. Once *S. aureus* reaches the stationary phase, most of the PG found in its membrane is converted to CL [55]. As mentioned above, this is done by the CL synthases Cls1 and Cls2 by condensing two PG molecules to create one CL and glycerol [55]. Cls2 primarily synthesises cardiolipin under normal conditions whereas Cls1 does so under acid stress [54]. Cardiolipins can stabilise liposomes against osmotic stress and have been shown to be unnecessary for growth under high salt conditions but essential for long term survival in that environment [63]. Cardiolipins have also been shown to contribute to resistance to daptomycin by preventing membrane permeabilisation [64].

Monoglycosyldiacylglycerols and diglycosyldiacylglycerols have been shown to be responsible for the stability and fluidity of the cell membrane by their relative proportion. This is due to MGDG being non-bilayer forming whereas DGDG is bilayer forming [57]. Living organisms need to be able to synthesise bilayer and non-bilayer forming lipids and adjust their relative ratios in order to respond to the effects of external triggering events [56]. Monoglycosyldiacylglycerol and diglycosyldiacylglycerol provide this function. In addition, the βGlc(1→6)βGlc-DAG serves as a membrane anchor for lipoteichoic acids in Gram-positive bacteria including *S. aureus* [56].

Generally, at neutral pH the membrane of *S. aureus* is made up of approximately 55% PG, 40% Lys-PG, and 5% CL [65]. However, the phospholipid composition of the membrane can be changed in response to changes in growth phase as well as changes in external stimuli such as osmolarity, pH and temperature [63]. This is done to change the properties of the membrane in order to help the cell survive. One such alteration can be made to the relative abundances of PG and Lys-PG. Phosphatidylglycerol is negatively charged whereas Lys-PG is positively charged, and one can be made from the other by the action of the enzyme MprF [54]. Doing so results in an increase of one or the other, making the membrane more negatively or positively charged. This can help bacteria resist the action of antibiotics such as CAMPs. As CAMPs are positively charged an increase in Lys-PG and decrease in PG leads to an increase in the membrane charge which helps to repel CAMPs [66]. Bacteria resistant to CAMPs have been found to have a higher proportion of Lys-PG in their membranes and an mprF deficient mutant was found to be highly sensitive to CAMPs [13,67]. The method of upregulating Lys-PG is first triggered by CAMPs activating a sensor histidine kinase of the ApsSR regulon [65]. This then leads to the upregulation of mprF expression leading to an increase in the MprF protein product that catalyses the production of Lys-PG from PG, as mentioned above [65].

Changes in individual species of membrane lipids between MRSA and MSSA were identified in a previous study [45]. Three lipid species were identified to be significantly different between the tested MSSA and MRSA strains (a DGDG and two Lys-PG’s). These lipids were present at a higher abundance in the MSSA strains and indicate that there are differences in the cells beyond the well characterized methicillin resistance mechanism. 

### 6.3. Virulence Factors

The success of *S. aureus* as a pathogen is in part due to its extensive range of virulence factors. These virulence factors often possess more than one pathogenesis function and there are often multiple virulence factors with the same function [68]. Some of the common virulence factors found in *S. aureus* include phenol soluble modulins (PSMs), α-toxin, protein A, Panton–Valentine leucocidin, staphylococcal enterotoxins (SE), staphyloxanthin, arginine catabolic mobile element (ACME), coagulase, toxic shock syndrome toxin-1, exfoliative toxins and haemolysins [5]. The function of *S. aureus* virulence factors can be broken down into three main groups. These are the adhesins that allow bacteria to attach to human tissue, the toxins that cause tissue damage and the immunomodulators that interfere with host immunity (Table 3) [5]. 

Panton–Valentine leucocidin is a bicomponent toxin made up of the two subunits LukS and LukF which are encoded by *lukS-PV* and *lukF-PV* respectively [15]. It can be found in both MSSA and MRSA but is absent in most of the predominant HA-MRSA strains [15]. Panton–Valentine leucocidin is a pore forming toxin that targets immune system cells such as neutrophils and monocytes [69]. It also possesses pro-inflammatory effects. In order to lyse the cell, the LukS component must bind to the human complement receptors C5aR and C5L2 [69]. This allows for LukF to dock and for the two subunits to oligomerise into a pore, lysing the cell [69].

Phenol-soluble modulins are a family of amphipathic α-helical peptides [15]. They have cytolytic activity against a variety of cells including neutrophils and erythrocytes [15]. Phenol-soluble modulins are grouped by size and charge with PSMαs being shorter (20–25 amino acids) and PSMβs being larger (43–45 amino acids) [69]. PSMαs have a neutral or positive charge while PSMβs have a negative charge. PSMαs are more cytolytic and can trigger the inflammatory response through recruitment of neutrophils. PSMβs are less cytolytic but have been implicated in the spread of biofilms [69]. As PSMs are coded by genes in the core genome rather than in mobile genetic elements, they are found in nearly all strains of *S. aureus* [69]. 

Staphylococcal enterotoxins (SE), also known as superantigens, are a group of at least 27 members that includes both SE and staphylococcal enterotoxin-like (SE*l*) proteins [70]. SEs and SE*l*s differ in that SEs are capable of exerting emetic activity while SE*l*s cannot [70]. SEs exert their effect by binding to MHC class II receptors on T-cells [71]. This results in hyperstimulation and leads to rapid proliferation and massive cytokine release. This can lead to food poisoning and toxic shock syndrome [72]. Toxic shock syndrome toxin-1 is another superantigenic toxin similar to staphylococcal enterotoxins. It is coded by the *tst* gene and serves as a major virulence factor in toxic shock syndrome [71]. 

There are three exfoliative toxins in *S. aureus* called exfoliative toxin A, B and D [71]. Exfoliative toxins act as proteases that cleave desmoglein 1 that connects epidermal cells. As a result, these toxins are associated with exfoliation of the epidermis. This is done without necrolysis or inflammation [71]. Exfoliative toxins have also been implicated in staphylococcal scalded-skin syndrome [73].

There are three main haemolysins found in *S. aureus*. These are α-haemolysin, β- haemolysin and γ-haemolysin [74,75]. Both α- and γ-haemolysins are pore forming toxins that target a variety of human cells. α-haemolysin causes the destruction of epithelial cells, erythrocytes, monocytes and fibroblasts [74] while γ-haemolysin causes the destruction of neutrophils, monocytes and macrophages [75]. β-haemolysin is not a pore-forming toxin but rather a neutral sphingomyelinase that hydrolyses sphingomyelin causing lysis of red blood cells [75]. 

Protein A is a cell wall anchored protein expressed on nearly all strains of *S. aureus* [69]. It is encoded by the *spa* gene and binds to the Fc region of IgG antibodies and to the Fab of Variable Heavy 3 idiotype B-cell receptors [69]. By binding IgG in this way, protein A impairs the host response in phagocytosis and clearing of *S. aureus*. It has also been shown to initiate the pro-inflammatory cascade in the airway [15]. Protein A binding to B-cells also induces rapid activation and expansion which is followed by apoptotic cell death [69]. In these ways protein A is able to contribute to impairment of the host defence system that assists in *S. aureus* colonization [15,69].

Staphyloxanthin is the pigment responsible for the golden colour associated with *S. aureus* [76]. Its role as a virulence factor is as an antioxidant to protect the cell from the reactive oxygen species generated by the host immune system [76]. Therefore, unlike the toxins described above, staphyloxanthin contributes to virulence by protecting the bacterial cell from the host and allowing for colonisation and spread rather than by targeting the host directly [76].

The ACME is another virulence factor that contributes to virulence by assisting with colonisation rather than by targeting host cells [74,77]. It is a 30.9 kb segment of DNA unique to the USA300 MRSA strain [74]. It includes a cluster of *arc* genes that encode an arginine deiminase pathway and the oligopeptide permease operon [74]. The arginine deiminase pathway can convert L-arginine to carbon dioxide, ATP, and ammonia [77]. It is thought that this contributes to *S. aureus* colonisation by ammonification of the acidic skin environment [15]. 

Coagulases also contribute to *S. aureus* virulence by assisting in colonisation and evasion of the immune system rather than by targeting host cells [78,79]. There are two coagulases in *S. aureus* that contribute to its virulence. These are named coagulase (Coa) and von Willebrand factor binding protein (vWbp) [79]. During infection, both coagulases trigger the cleavage of fibrinogen to fibrin [78]. It is believed that the fibrin is then able to coat the surface of *S. aureus,* thereby allowing it to evade the opsonophagocytic clearance of the host immune system [79].

**Table 3:** Virulence factors present in *S. aureus*.

Virulence Factor	Group	Function	Reference
Panton–Valentine Leucocidin	Toxin	Pore forming toxin that targets neutrophils and monocytes.	[69]
Phenol-Soluble Modulin	Toxin	Cytolytic activity against neutrophils and erythrocytes.	[15]
Staphylococcal Enterotoxin	Toxin	Binds to MHC class II receptors on T-cells causing rapid proliferation and massive cytokine release.	[72]
Toxic Shock Syndrome Toxin-1	Toxin	Major virulence factor in toxic shock syndrome.	[71]
Exfoliative Toxin	Toxin	Protease that cleaves desmoglein 1 that connects epidermal cells.	[71,73]
Haemolysin	Toxin	Pore forming toxin that causes destruction of epithelial cells, erythrocytes, monocytes, fibroblasts, neutrophils and macrophages.	[74,75]
Protein A	Immunomodulator	Cell wall anchored protein that binds to IgG antibodies, impairing host response in phagocytosis.	[15,69]
Staphyloxanthin	Immunomodulator	Protects cell from reactive oxygen species produced by the host immune system.	[76]
Arginine Catabolic Mobile Element	Immunomodulator	Contributes to colonisation by ammonification of the acidic skin environment.	[15,77]
Coagulase	Immunomodulator	Triggers cleavage of fibrinogen to fibrin that coats cell surface allowing evasion of opsonophagocytic clearance by the host immune system.	[78,79]

#### Therapy Targeting Virulence Factors in *Staphylococcus aureus*

Emergence of antibiotic resistance in *S aureus* is outpacing the discovery of new antibiotics for treating infections [80]. Finding adjuvants to antibiotic therapy is promising because they can allow treatment of infections from resistant strains with antibiotics that are already in practice. Studies reporting the potential of anti-virulence strategies as adjuvants to antibiotics against *S aureus* have increased in the past decade [81,82,83,84]. There are many advantages of using anti-virulence adjuvants. For example, bacteria are less likely to develop resistance to them as anti-virulence compounds do not affect the viability of bacteria and target non-essential genes [85]. This results in a reduced selective pressure with low possibility of development of resistance [85]. Furthermore, since anti-virulence compounds do not target bacterial viability, they do not affect the commensal flora of the host which would be beneficial for patient health during and after treatment [86]. Finally, anti-virulence drugs provide new therapeutic targets such as those aimed at pore-forming toxins, immune evasion mechanism and quorum sensing system [82]. This is especially beneficial for bacteria such as *S. aureus* in which there is resistance to many commonly used antibiotics that target traditional bacterial components. 

## 7. Conclusions

*Staphylococcus aureus* is a significant threat to human health that has been made worse by its continued development of antibiotic resistance. Novel antimicrobials and treatment options are needed to combat this but there has been difficulty in developing such methods. A deeper understanding of common antibiotic targets such as the cell wall and cell membrane and how these change in antibiotic resistant strains can provide insight when developing novel antibiotics. Furthermore, understanding virulence factors that contribute to *S. aureus’* success as a human pathogen would assist in development of novel adjuvant therapies. 

## Figures and Tables

**Table 1:** Characteristics of healthcare-associated, community-associated and livestock-associated methicillin resistant *S. aureus*.

Type of MRSA	Location Acquired	Associated Diseases
Healthcare-Associated	Obtained in healthcare settings including nursing homes and hospitals	Bloodstream infections, pneumonia
Community-Associated	Obtained in the wider community or within 48 h of admission to a hospital	Skin and soft tissue infections
Livestock-Associated	Obtained from close contact with livestock such as cows, pigs and chickens	Mainly skin and soft tissue infections

**Table 2:** Lipid classes present in the bacterial cell membrane, their role in the cell and their role in antibiotic resistance.

Lipid Class	Role in Cell Functioning	Role in Antibiotic Resistance	Reference
Phosphatidylglycerol	Contributes negative charge to membrane. Functions as stabiliser and destabiliser by electrostatic interactions between charged species.	Increases susceptibility to positively charged molecules such as CAMPs.	[59,60]
Lysyl-Phosphatidylglycerol	Contributes positive charge to membrane. Regulates DNA replication initiation through DNA replication initiator protein DnaA.	Decreases susceptibility to positively charged molecules such as CAMPs.	[54,61]
Cardiolipin	Stabilises liposomes against osmotic stress. Essential for long term survival in high salt conditions.	Contributes to daptomycin resistance by preventing membrane permeabilisation	[62,63,64]
Monoglycosyldiacylglycerol	Responsible for the stability and fluidity of the cell membrane. Non-bilayer forming.	May play a role in affecting the permeability of the cell to antibiotics.	[57]
Diglycosyldiacylglycerol	Responsible for the stability and fluidity of the cell membrane. Bilayer forming.	May play a role in affecting the permeability of the cell to antibiotics.	[57]

## Data Availability

Not applicable.
